# A review of the genera *Gnathochorisis* Förster and *Symplecis* Förster of South Korea, with notes on Korean orthocentrines (Hymenoptera, Ichneumonidae, Orthocentrinae)

**DOI:** 10.3897/zookeys.562.7303

**Published:** 2016-02-10

**Authors:** Andrei E. Humala, Jin-Kyung Choi, Jong-Wook Lee

**Affiliations:** 1 Forest Research Institute, Russian Academy of Sciences, Petrozavodsk, Russia; 2 Department of Life Sciences, Yeungnam University, Gyeongsan, South Korea

**Keywords:** Fauna, ichneumon wasps, keys, Korea, new species, taxonomy

## Abstract

Two genera of Korean Orthocentrinae, *Gnathochorisis* and *Symplecis*, are reviewed, and keys to species of these genera are provided here. Two new species, *Gnathochorisis fuscipes* Humala & Lee, **sp. n.** and *Gnathochorisis koreensis* Humala & Lee, **sp. n.** are described from South Korea. The current state of the taxonomy of Eastern Palaearctic orthocentrines is briefly discussed.

## Introduction

Orthocentrinae is a moderately large, cosmopolitan subfamily of small-bodied ichneumon wasps consisting of approximately 500 described species (Yu et al. 2012). Most orthocentrines are koinobiont endoparasitoids of nematoceran Diptera (Sciaroidea), the larvae of which often develop in fungal fruiting bodies (Roman 1923, Askew and Shaw 1986, Humala 2003). Only Orthocentrinae *sensu stricto* (Townes 1971), or the *Orthocentrus* genus-group (Wahl and Gauld 1998), is morphologically well defined, comprising a distinctive, monophyletic lineage within the subfamily (Broad 2010), while the remaining genera–for a long time considered a “wastebasket” group among ichneumon wasps, which is the most difficult of the ichneumonid subfamilies to define (Townes 1971)–have significant morphological diversity. The current concept of Orthocentrinae includes most of the genera comprising Townes’s Orthocentrinae and Microleptinae (Helictinae *auct*.) with the exception of some genera. A key to the genera was provided by Townes (1971), but it contained several genera (*Tatogaster* Townes, *Acaenitellus* Morley, *Microleptes* Gravenhorst, *Oxytorus* Förster, *Cylloceria* Schiødte and *Allomacrus* Förster) which were excluded afterwards (Wahl 1986, 1990, Gupta 1988, Wahl and Gauld 1998). Several other genera described by Rossem–*Pantomima* Rossem, *Fetialis* Rossem, *Epitropus* Rossem and *Phosphoriana* Rossem–were synonymized later (Broad 2004, Humala 2007), and *Hyperacmus* Holmgren was transferred to the Cylloceriinae by Quicke et al. (2009).

The subfamily Orthocentrinae remains one of the least known among ichneumonid wasps, and even the European fauna remains relatively unknown. Many genera within this subfamily are in need of modern revisions and potential reclassification, as emphasized by Broad (2010). The Nearctic fauna (excluding Orthocentrinae *s. str.*) was revised by Dasch (1992), the Western Palaearctic fauna was revised by Aubert (1968, 1976, 1977, 1978, 1980, 1981) and Rossem (1981, 1983, 1987, 1988, 1990, 1991, etc.), whereas the Eastern Palaearctic is scarcely covered by taxonomic and faunistic studies (Uchida 1930, 1942; Rossem 1981-1991; Humala 2003, 2007; Kasparyan et al. 2012, *etc.*). Other regions have not been practically studied yet, with the exception of the Neotropics, where a partial revision of the genus *Orthocentrus* Gravenhorst was recently published (Veijalainen et al. 2014; Zwakhals and Diller 2015).

Two genera of Orthocentrinae in the fauna of South Korea are treated here: *Gnathochorisis* with five species (two of them new to science) and *Symplecis* with two species. Keys to species occurring in South Korea are provided. This paper is the first dealing with orthocentrine ichneumon wasps occurring in South Korea.

## Materials and methods

Materials used in this study were collected by sweep nets and Malaise traps, after which they were deposited in the Animal Systematic Laboratory of Yeungnam University (YNU, Gyeongsan, South Korea). Photographs were taken using an AxioCam MRc5 camera attached to a stereo microscope (Zeiss SteREO Discovery. V20), processed using AxioVision SE64 software (Carl Zeiss), and optimized with a Delta imaging system (i-solution, IMT i-Solution Inc.); and a Leica DFC 290 digital camera attached to a Leica MZ9.5 stereomicroscope; images were combined using Helicon Focus Pro software.

The morphological terminology mostly follows Gauld (1991). Note that we use the convenient term ‘temple’ for the upper part of the gena, between the eye and the occipital carina.

**Abbreviations are used as follows**:

GW
 Gangwon-do 


GG
 Gyeonggi-do 


CB
 Chungcheongbuk-do 


CN
 Chungcheonnam-do 


GB
 Gyeongsangbuk-do 


GN
 Gyeongsangnam-do 


AEI
American Entomological Institute, Gainesville, Florida, U.S.A. (H. Townes collection) 


DEI
Deutsches Entomologisches Institut, Eberswalde, Germany 


IZU
 Instytut Zoologiczny Uniwersytetu, Wroclaw, Poland (Gravenhorst collection) 


NM
Naturhistorisches Museum, Wien, Austria 


ZI
 Zoologiska Institutionen, Lund, Sweden 


## Results

### Family Ichneumonidae Latreille, 1802 Subfamily Orthocentrinae Förster, 1869

#### Genus Gnathochorisis

Taxon classificationAnimaliaHymenopteraIchneumonidae

Förster, 1869

Gnathochorisis Förster, 1869: 152. Type species: *Gnathochorisis flavipes* Förster, 1871: 113.
Blapticus Förster, 1869: 171. Type species: *Blapticus leucostomus* Förster, 1871: 83.
Laepserus Förster, 1869: 205. Type species: *Blapticus crassulus* Thomson, 1888: 1289.
Acroblapticus Schmiedeknecht, 1911: 2173. Type species: *Blapticus dentifer* Thomson, 1888: 1288.

##### Diagnosis.

Body rather stout. Head transverse; clypeus small, weakly to strongly separated from face by a groove; eye large; temple short; malar space with subocular sulcus; occipital carina complete; antenna long, male antenna lacking tyloids. Mesosoma finely or densely punctate on mesoscutum, polished on mesopleuron. Epicnemial carina complete, dorsally distant from anterior margin of mesopleuron; propodeum polished or matt, usually with carinae complete and strong; often propodeal apophyses somewhat developed. Fore wing with areolet present or absent, when present sessile or short petiolate, rectangular. Hind leg as a rule stout, hind femur 2.85–4.9 times as long as high. First metasomal segment with glymma lacking, sternite fused to tergite and reaching 0.5–0.6 of the segment, with spiracle near middle; second tergite matt or polished, sometimes with longitudinal striae; ovipositor upcurved, with a dorsal subapical notch, 0.5–1.1 times as long as hind tibia.

##### Remarks.

Medium-sized genus, with 13 described species (Yu et al. 2012). Eight species occur in the Nearctic region and seven in the Palaearctic region (two species are distributed on both continents). Beyond the Holarctic region, one species of *Gnathochorisis* is known in Mexico (Dasch 1992, Humala et al. 2011). The genus was also reported from Australia (Gauld 1984), Ecuador, and Central America (Veijalainen et al. 2012). Five species of *Gnathochorisis*, including two newly described, are presently reported from South Korea here. This is the first record of the genus from this country. In the European part of Russia *Gnathochorisis flavipes* Förster was reared from the fungus gnat *Neoempheria striata* Meigen (Mycetophilidae: Mycomyinae) (Humala 2003), other published host records (Dasch 1992) seem to be doubtful.

##### Key to species of *Gnathochorisis* occurring in South Korea

**Table:** 

1	Fore wing with areolet (Figs 11, 16, 21). Sculpture of the second tergite various	**2**
–	Fore wing without areolet (Figs 4, 28). Second tergite of metasoma polished, longitudinally striate	**4**
2	Metapleuron coriaceous (Fig. 22); notauli short, developed in anterior 0.3 of mesoscutum. Second tergite coriaceous (Figs 23, 24). Hind femur not strongly inflated, 4.0–4.1 times as long as wide	***Gnathochorisis dentifer* Thomson**
–	Metapleuron polished and impunctate (Figs 17, 27). Hind femur stout, 3.0–3.8 times as long as high	**3**
3	Second tergite coriaceous, without longitudinal striae (Fig. 18). Female frontal orbits with yellow or pale marks (Fig. 15); notauli developed only in anterior third of mesoscutum. Hind femur 3.5–3.8 times as long as high	***Gnathochorisis crassulus* Thomson**
–	Second tergite polished, longitudinally striate (Fig. 12). Female frontal orbits fuscous (Fig. 8); notauli well developed, meeting in the centre of mesoscutum (Fig. 9). Hind femur inflated, 3.0 times as long as wide	***Gnathochorisis koreensis* Humala & Lee, sp. n.**
4	Female face at the level of antennal fossae 0.47 times as wide as head; face brown near antennal sockets (Fig. 26). Postocellar line equal to maximum diameter of the lateral ocellus. Hind coxa and femur yellow (Fig. 25). Second tergite with yellowish apical band (Fig. 29)	***Gnathochorisis flavipes* Förster**
–	Female face at the level of antennal fossae 0.53 times as wide as head, face entirely black (Fig. 2). Postocellar line 1.3 times as long as maximum diameter of the lateral ocellus. Hind coxa and femur infuscate (Fig. 1). Second tergite entirely fuscous	***Gnathochorisis fuscipes* Humala & Lee, sp. n.**

**Figures 1–6.**
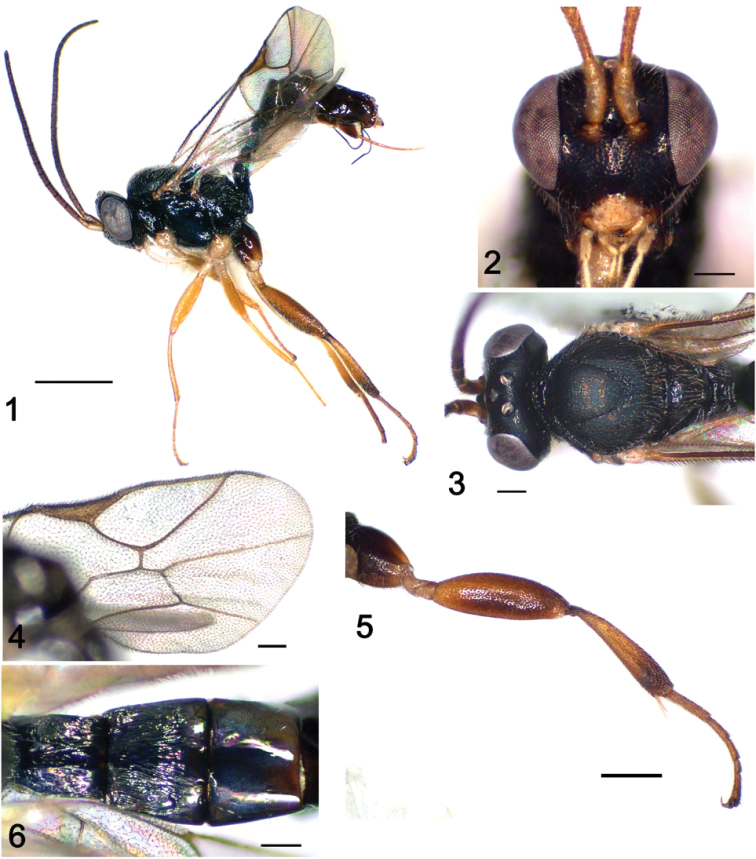
*Gnathochorisis fuscipes* sp. n. (holotype, female); **1** Habitus, lateral view **2** Head and base of antenna, anterior view **3** Head and mesoscutum, dorsal view **4** Fore wing **5** Hind leg, lateral view **6** Metasoma basal tergites, dorsal view. Scale bars: 1.0 mm (**1**); 0.5 mm (**5**); 0.2 mm (**2–4, 6**).

**Figures 7–13.**
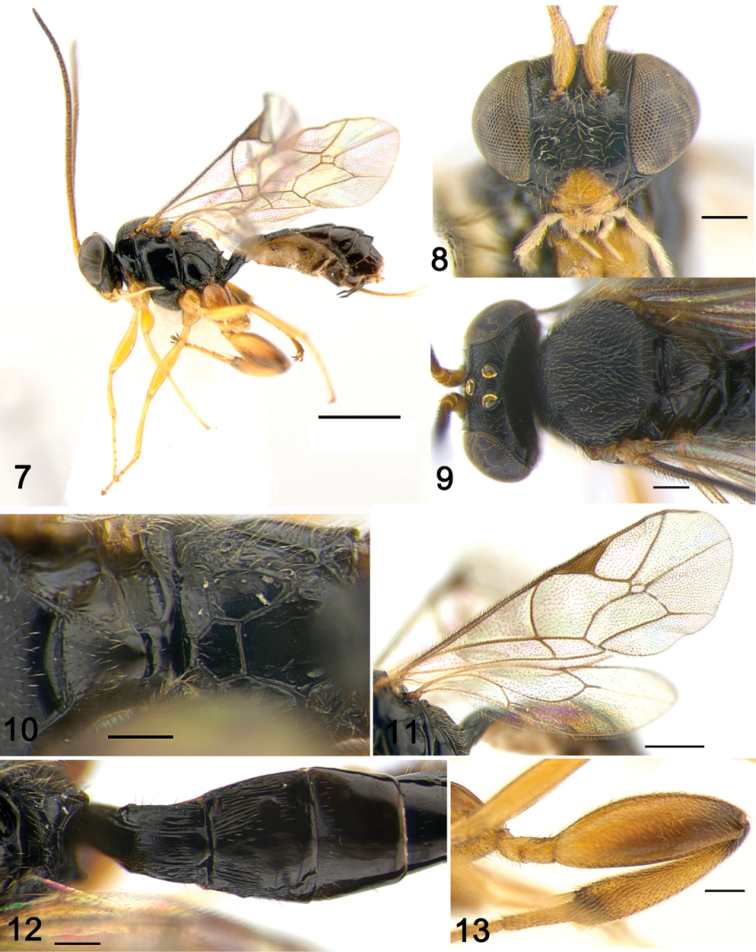
*Gnathochorisis koreensis* sp. n. (holotype, female); **7** Habitus, lateral view **8** Head, anterior view **9** Head and mesoscutum, dorsal view **10** Propodeum, dorsal view **11** Wings **12** Metasoma, basal tergites, dorsal view **13** Hind femur, lateral view. Scale bars: 1.0 mm (**7**); 0.5 mm (**5**); 0.2 mm (**8–10, 12, 13**).

**Figures 14–18.**
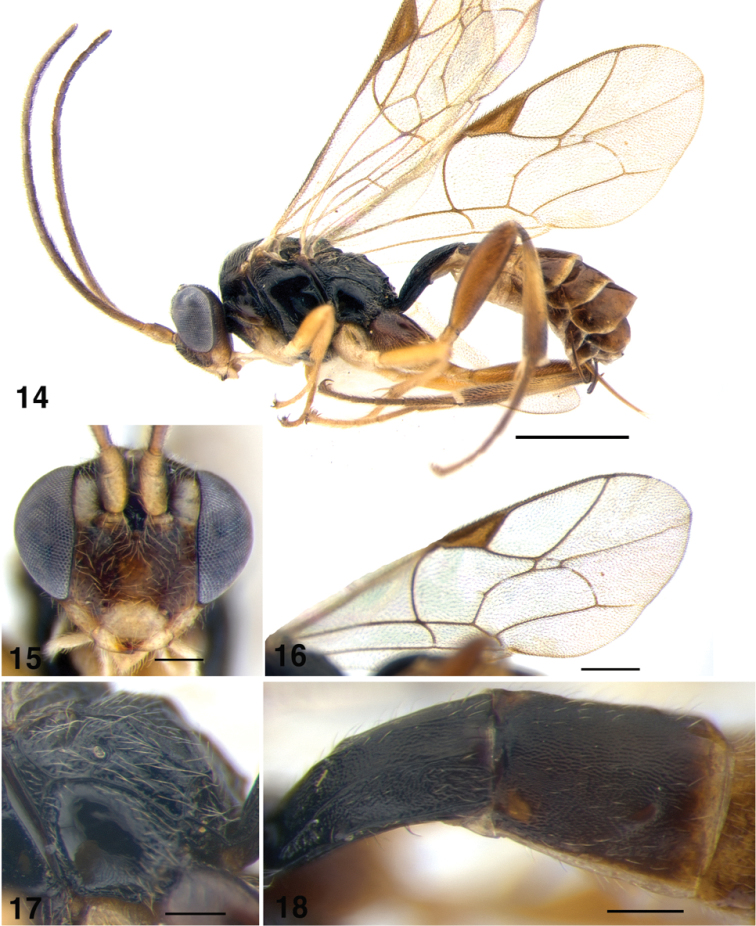
*Gnathochorisis crassulus*; **14** Habitus, lateral view; **15** Head of female, anterior view **16** Fore wing **17** Metapleuron, lateral view **18** Metasoma, first and second tergites, dorsolateral view. Scale bars: 1 mm (**14**); 0.5 mm (**16**); 0.2 mm (**15, 17, 18**).

**Figures 19–24.**
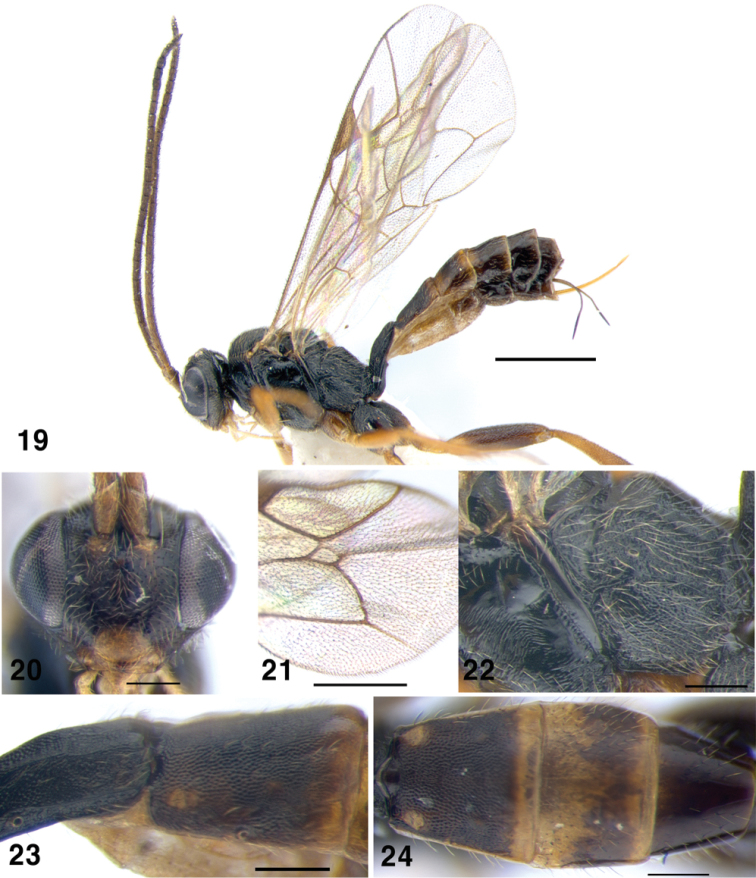
*Gnathochorisis dentifer*; **19** Habitus, lateral view **20** Head of female, anterior view **21** Areolet of fore wing **22** Metapleuron, lateral view **23** Metasoma, second tergite, lateral view; **24** Metasoma, second to fourth tergites, dorsal view. Scale bars: 1 mm (**19**); 0.5 mm (**21**); 0.2 mm (**20, 22–24**).

**Figures 25–29.**
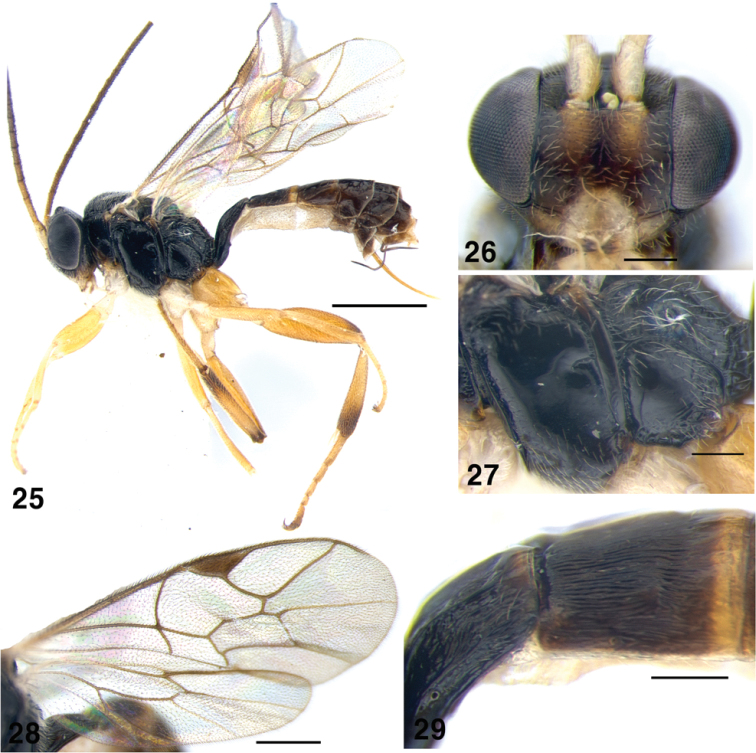
*Gnathochorisis flavipes*; **25** Habitus, lateral view **26** Head of female, anterior view **27** Metapleuron, lateral view **28** Wings **29** Metasoma, second tergite, lateral view. Scale bars: 1 mm (**25**); 0.5 mm (**28**); 0.2 mm (**26, 27, 29**).

#### Gnathochorisis fuscipes

Taxon classificationAnimaliaHymenopteraIchneumonidae

Humala & Lee sp. n.

http://zoobank.org/C11D8868-47AF-4B82-B629-859C4B334027

Figs 1–6

##### Diagnosis.

Closely allied to *Gnathochorisis flavipes* Grav. but differs by its wider and matt face, more matt and rough sculpture of the mesoscutum, the slenderer first and second tergites of metasoma; the absence of light apical bands on tergites 2–3, the presence of sclerotized area on second sternite, the stouter flagellomeres, the fuscous hind coxa, and the hind tibia infuscate apically and in apical third. Separable from other known Palaearctic *Gnathochorisis* species by the absence of a closed areolet in the fore wing.

##### Description.

**Female** (holotype). Fore wing length 3.8 mm.

***Head*.** 1.1 times as wide as high; frons nearly polished with weak microsculpture; face polished with sparse and fine punctures, at the level of antennal fossae 0.5 times as wide as head (Fig. 2). Inner eye orbits slightly divergent ventrally. Clypeus weakly separated from face, approximately 1.9 times as wide as high, edge of clypeus convex; malar space 1.8 times as long as mandible basal width, with subocular sulcus; maxillary palp reaching beyond fore coxa. In dorsal view, head posteriorly deeply concave; occipital carina complete; temples short; ocular-ocellar line 1.3 times as long as maximum diameter of lateral ocellus, equal to postocellar line (Fig. 3). Antenna moderately long, with 21 flagellomeres, basal flagellomere 3.9 times and second flagellomere 3.0 times as long as wide.

***Mesosoma*.** 1.4 times as long as high. Mesoscutum matt with short adpressed dense setae; notauli well developed, meeting in the centre of mesoscutum (Fig. 3); epicnemial carina complete; in profile, scutellum somewhat high, without lateral carinae. Propodeum polished with sparse setae; anterior transverse carina strongly raised; area superomedia transverse, costula present; rounded apophyses of propodeum resulting from crossing lateral longitudinal and posterior transverse carinae developed. Spiracle of moderate size. Most of metapleuron polished with small coriaceous area near base of hind coxa. Fore wing without areolet, with 2rs-m shorter than second abscissa of 1m-cu (Fig. 4); cu-a inclivous, slightly postfurcal. Hind wing with first abscissa of Cu1 inclivous, 2 times as long as cu-a, distal abscissa of Cu1 present. Hind leg stout, coxa and femur polished, tibia and tarsus coriaceous, hind femur inflated, 3.2 times as long as high (Fig. 5). Hind tibia 4.8 times as long as its maximum width, with spine-like setae; hind basitarsus 0.4 times as long as hind tibia.

***Metasoma*.** First metasomal segment moderately arched, 2.2 times as long as its posterior width, polished, with dorsal longitudinal carina strong; postpetiole longitudinally striate. Spiracle at 0.65, sternite at 0.55 of tergite 1 length. Second tergite 0.85 times as long as its posterior width, with small thyridium basally; polished with longitudinal striae, restricted by transverse groove at apical 0.25 (Fig. 6). Remainder tergites polished, metasoma somewhat compressed laterally from tergite 3. Ovipositor upcurved, approximately as long as first metasomal segment, tip with blunt subapical dorsal notch.

**Colour.** Fuscous. Clypeus, mandible, palpi, tegula, corners of pronotum, wings bases, antenna ventrally, except for brownish flagellomeres in apical 1/3 of flagellum, pale yellow. Legs basically light brown, hind coxa dark brown basally, hind tibia somewhat darkened basally and apically, fore and middle coxae and all trochanters pale yellow. Metasoma from apical third of tergite 3 brown. Wings hyaline, veins and pterostigma brown.

**Male.** Unknown.

##### Etymology.

Named after the fuscous hind legs.

##### Material examined.

Holotype: female (YNU), Korea: GW, Wonju-si, Socho-myeon, Hakgong-ri, Chiaksan National Park, 37°22'18"N, 128°03'1.84"E, Malaise trap, 9–20 June 2013 (J.W. Lee)

##### Distribution.

South Korea (GW).

#### Gnathochorisis koreensis

Taxon classificationAnimaliaHymenopteraIchneumonidae

Humala & Lee sp. n.

http://zoobank.org/49D8B9C9-43D8-4AC9-8800-07B01A46C488

Figs 7–13

##### Diagnosis.

Fore wing with areolet. Metapleuron polished; notauli well developed, meeting in the centre of mesoscutum. Female frontal orbits fuscous. Second tergite polished, longitudinally striate. Hind femur inflated, 3.0 times as long as wide. From similar Palaearctic *Gnathochorisis flavipes* Grav. it differs by the presence of closed areolet and the absence of light apical bands on tergites 2–3. Separable from other known Palaearctic *Gnathochorisis* species with closed areolet by the polished metapleuron, fine sculpture of mesoscutum, long notauli meeting in the centre of mesoscutum, narrow face, entirely fuscous and polished metasomal tergites with longitudinal striae on tergite 2. From the more closely allied *Gnathochorisis meridionator* Aubert, reported from Russian Far East (Humala 2007), it differs by the wide and entirely fuscous face, strong, distinct notauli meeting in the centre of the mesoscutum, short metasomal tergites, infuscate hind coxae, and stouter antennae.

##### Description.

**Female** (holotype). Fore wing length 3.3 mm.

***Head*.** 1.2 times as wide as high; frons nearly polished with weak microsculpture; face polished, sparsely and finely punctate, at the level of antennal fossae 0.45 times as wide as head (Fig. 8); inner eye orbits subparallel. Clypeus weakly separated from face, approximately 1.8 times as wide as high, edge of clypeus convex; temples short; ocular-ocellar line 1.3 times as long as maximum diameter of lateral ocellus, postocellar line 0.8 times as long as maximum diameter of lateral ocellus (Fig. 9). Antenna moderately long, with 20 (21 in paratype) elongate flagellomeres; basal flagellomere 4.5 times and second flagellomere 3.6 times as high as wide.

***Mesosoma*.** 1.45 times as long as high. Mesoscutum convex, matt with short adpressed dense setae; epomia present; notauli well developed, meeting in centre of mesoscutum (Fig. 9); epicnemial carina complete; in profile scutellum somewhat high, with lateral carinae anteriorly. Most of mesopleuron and metapleuron polished. Propodeum polished with sparse setae; carinae complete and strong; area superomedia slightly transverse (Fig. 10); small propodeal apophyses present; spiracle small. Fore wing with areolet closed, small, short petiolate and slightly longer than high; 3rs-m shorter than 2rs-m (Fig. 11); cu-a inclivous, nearly interstitial. Hind wing with first abscissa of Cu1 inclivous, 2 times as long as cu-a, distal abscissa of Cu1 present but weakly pigmented. Hind leg stout, coxa and femur polished, tibia and tarsus coriaceous; femur inflated, 3.0 times as long as high (Fig. 12); tibia 4.8 times as long as maximum width subapically, with spine-like setae and dense fringe on apex well developed; hind basitarsus 0.35 times as long as hind tibia.

***Metasoma*.** First metasomal segment moderately arched, 2.1 times as long as its posterior width, dorsal longitudinal carina well developed; postpetiole polished, striate laterally; spiracle and end of sternite near middle of tergite length. Second tergite 0.85 times as long as its posterior width, polished with small thyridium basally and oblique longitudinal striae basally and laterally, whereas central and apical parts smooth (Fig. 13). Remaining tergites of metasoma polished, somewhat compressed laterally from tergite 3. Ovipositor upcurved, approximately 0.7 times as long as hind tibia.

**Colour.** Fuscous. Clypeus, mandible, tegula, wings bases, dorsal corner of pronotum, antenna ventrally yellow, palp whitish yellow. Legs mostly light brown, hind coxa reddish brown, darkened basally, hind femur gradually infuscate to apex, hind tibia somewhat darkened basally and apically. Metasoma fuscous; tergites 2–4 with reddish brown apical bands; thyridium reddish brown. Wings hyaline, veins and pterostigma brown.

**Male.** Unknown.

##### Etymology.

Named after the type locality, Korea.

##### Material examined.

Holotype female (YNU), Korea: GB, Mungyeong-si, Geaun-eup Wanjang-ri, Songnisan National Park, Beorimigijae, 36°40'59"N, 127°57'07"E, Malaise trap, 12 August–11 September 2013 (J.K. Choi). Paratype female (YNU), Korea: GG, Kwangju-si, Docheong-myeon Mt. Taehwasan, Malaise trap, 15–25 June 2008 (J.K. Choi)

##### Distribution.

South Korea (GG).

#### Gnathochorisis crassulus

Taxon classificationAnimaliaHymenopteraIchneumonidae

(Thomson, 1888)

Figs 14–18

Blapticus crassulus Thomson 1888; type depository: ZI.
Acroblapticus crassulus Thomson (Schmiedeknecht, 1911).
Gnathochorisis crassulus Thomson (Aubert, 1966).

##### Diagnosis.

Inner eye orbits slightly divergent ventrally, face matt, finely punctate; antenna moderately long with 22–24 flagellomeres. Mesosoma finely and densely punctate on mesoscutum and mesosternum, polished on metapleuron. Propodeum polished, carinae complete and strong. Fore wing with areolet short petiolate, rectangular (Fig. 16). Hind femur stout, 3.5–3.8 times as long as high. First tergite with dorsolateral carina strong (Fig. 18); second tergite matt; ovipositor upcurved, 0.95 times as long as hind tibia. Fuscous; pale yellow on frontal orbits, malar space, clypeus, mandibles, palpi, scape and pedicel ventrally, tegula, wing bases, hind corner of pronotum, fore and middle coxae and all trochanters, apical margins of metasomal tergites 2–4. Male with yellow face, inner orbits and malar space; propleuron and lower mesopleuron yellowish.

##### Material examined.

Korea: 1 female, GW, Taebaek-si, Hyeol-dong, Yuilsa, 37°06'N 128°26'E, Malaise trap, 30 June 1991 (J.W. Lee).

##### Distribution.

Holarctic; in Palaearctic region it was reported from Europe, Siberia, Russian Far East (Humala 2003, 2007), Japan (Dasch 1992; no data on the examined material was provided), and South Korea (new record).

#### Gnathochorisis dentifer

Taxon classificationAnimaliaHymenopteraIchneumonidae

(Thomson, 1888)

Figs 19–24

Blapticus dentifer Thomson 1888: 1288; type depository: ZI.
Acroblapticus dentifer Thomson (Schmiedeknecht, 1911: 2174).
Acroblapticus debilis Schmiedeknecht 1911: 2175; type depository: NM.
Gnathochorisis dentifer Thomson (Aubert 1966: 115–134).

##### Diagnosis.

Inner eye orbits slightly divergent ventrally, face lightly matt; antenna moderately long with 22–27 flagellomeres. Mesosoma finely and densely punctate on mesoscutum, coriaceous on metapleurum. Propodeum matt, carinae complete. Fore wing with areolet short petiolate, rectangular (Fig. 21). Hind femur stout, 4.0–4.15 times as long as high. First tergite with dorsolateral carina strong; second tergite matt (Fig. 24); ovipositor 1.0–1.1 times as long as hind tibia. Fuscous, including frontal orbits; pale yellow on clypeus, mandibles, palpi, scape and pedicel ventrally, tegula, wing bases, hind corner of pronotum, fore and middle coxae and all trochanters. Rufous on rest of fore and middle legs, apical margins of metasomal tergites 2–4, and basal margins on tergites 3–6. Male with yellow face, inner orbits and malar space.

##### Material examined.

Korea: 1 female, GW, Donghae-si, Samhwa-dong, Mureung Valley, 37°27'N 129°01'E, Malaise trap, 16 October–25 November 2005 (J.W. Lee) (YNU).

##### Distribution.

Holarctic; in Palaearctic region it was reported from Europe, Siberia, Russian Far East (Humala 2003, 2007, etc.), Japan (Dasch 1992; no data on the examined material was provided), and South Korea (new record).

#### Gnathochorisis flavipes

Taxon classificationAnimaliaHymenopteraIchneumonidae

Förster, 1871

Figs 25–29

Gnathochorisis terebrata Strand 1918: 159; type depository: DEI.
Gnathochorisis flavipes Förster 1871: 113.
Blapticus (Gnathochorisis) flavipes Förster (Thomson 1888: 1291).
Gnathochorisis flavipes Förster (Schmiedeknecht 1911: 2181).

##### Diagnosis.

Female face at the level of antennal fossae 0.47 times as wide as head; clypeus rather small, weakly separated from face by a groove, flattened; maxillary palp long, almost reaching middle coxae; malar space with distinct subocular sulcus; antenna moderately long with 20–21 flagellomeres. Mesosoma slightly matt and finely punctate on mesoscutum, polished on pronotum, mesopleuron and metapleuron (Fig. 27). Notaulus reaching centre of mesoscutum. Propodeum polished, carinae complete and strong, propodeal apophyses present; area superomedia transverse. Fore wing areolet absent (Fig. 28). Hind leg stout, femur 2.8–3.3 times as long as high; second tergite polished with distinct longitudinal striae (Fig. 29), apical margin polished; ovipositor 0.5–0.6 times as long as hind tibia. Fuscous, pale on clypeus, mandible, palpi, scape and pedicel ventrally, tegula, wing bases, hind corner of pronotum, fore and middle coxae and trochanters. Apical margins of metasomal tergites 2 and 3 and rest of legs yellowish-rufous. Female face brown, paler close to antennal sockets, male with face, inner eye orbits, scape, and malar space yellow.

##### Material examined.

Korea: 2 females, GW, Wonju-si, Socho-myeon, Hakgong-ri, Mt. Chiaksan, 37°22'18"N, 128°03'1.84"E, Malaise trap, 1–22 August 2013 (J.W. Lee); 2 females, GW, Wonju-si, Heungeup-myeon, Maeji-ri, Yonsei Univ., 20 July–28 August 2013 (H.Y. Han); 1 female, GW, Donghae-si, Samhwa-dong, Mureung Valley, 37°27'52"N, 129°01'26"E, Malaise trap, 5–18 August 2007 (J.W. Lee); 1 female, GW, Donghae-si, Samhwa-dong, Mureung Valley, 10–20 September 2006 (J.W. Lee); 2 females, CB, Chungju-si, Suanbo-myeon, Samun-ri, Woraksan National Park, 35°49'46"N, 128°04'05"E, 17 July–12 August 2013 (J.K. Choi); 1 female, CN, Seosan-si, Haemi-myeon, Daedok-ri, Hanseo Univ., 16 July–3 August 2013 (J.K. Kim); 1 female, GB, Cheongdo-gun, Unmun-myeon, Unmunsan, 6 June–1 July 2008 (J.W. Lee); 1 female, GN, Jinju-si, Gajwa-dong, 11–18 November1987 (J.W. Lee); 1 female, GN, Jinju-si, Gajwa-dong, 12–18 August 1989 (J.W. Lee).

##### Distribution.

Palaearctic; reported from Europe, Siberia and Russian Far East (Primorsky Terr.) (Humala 2003, 2007) and South Korea (new record).

##### Biology.

Reared from *Neoempheria striata* Mg. (Mycetophilidae) (Humala 2003).

#### Genus Symplecis

Taxon classificationAnimaliaHymenopteraIchneumonidae

Förster, 1869

Symplecis Förster 1869: 151. Type species: *Symplecis alpicola* Förster, 1871: 119; Thomson 1888: 1285; Schmiedeknecht 1911: 2169.
Blapticus Förster 1869: 171. Type species: *Blapticus leucostomus* Förster, 1871: 83.

##### Diagnosis.

Inner eye orbits strongly convergent ventrally in female, slightly in male; clypeus small, weakly to more strongly separated from face by a groove; eye large; temple short; malar space very narrow with subocular sulcus; mandible small, usually not twisted; male antenna lacking tyloids. Notaulus short and deep; epicnemial carina complete; carinae of propodeum complete and strong. Fore wing with areolet present or absent, when present sessile or short petiolate, rectangular. First metasomal segment slender, with glymma lacking, its sternite fused to tergite. Second tergite coriaceous, or with longitudinal striae. Ovipositor usually short, almost straight, stout at base, slenderer in apical part, 0.4–0.9 times as long as hind tibia.

##### Remarks.

Medium sized genus with 14 recognized species distributed worldwide: 11 species are known in the Palaearctic region, six in the Nearctic region (Dasch 1992), one in the Neotropical region, one in the Afrotropical region and one in the Oriental region (Yu et al. 2012).

Two species are reported from South Korea in this paper. This is the first record of the genus from this country. Both Korean species of *Symplecis* are Holarctic.

There are two known host records for *Symplecis*, both from Diptera, Sciaroidea: *Symplecis breviuscula* Roman was reared from *Diadocidia ferruginosa* Meigen (Diadocidiidae) in Europe (Roman 1923) and *Symplecis matilei* Delobel from *Neoempheria ombrophila* Matile et Matile (Mycetophilidae) in Central Africa (Delobel and Matile 1976).

##### Key to species of *Symplecis* occurring in South Korea

**Table:** 

1	Fore wing with areolet (Fig. 33). Ovipositor short, hardly surpassing apex of metasoma (Fig. 34). Second tergite of metasoma longitudinally striate (Fig. 35). Inner eye orbits strongly convergent ventrally in female (Fig. 31), slightly convergent in male (Fig. 32). Area superomedia of propodeum slightly transverse	***Symplecis bicingulata* Gravenhorst**
–	Fore wing without areolet (vein 3rs-m absent) (Fig. 39). Ovipositor long, 0.8–0.95 times as long as hind tibia. First and second tergites of metasoma coriaceous (Fig. 41). Inner eye orbits strongly convergent ventrally in both sexes (Figs 37, 38). Area superomedia of propodeum longer than wide	***Symplecis invisitata* Rossem**

**Figures 30–35.**
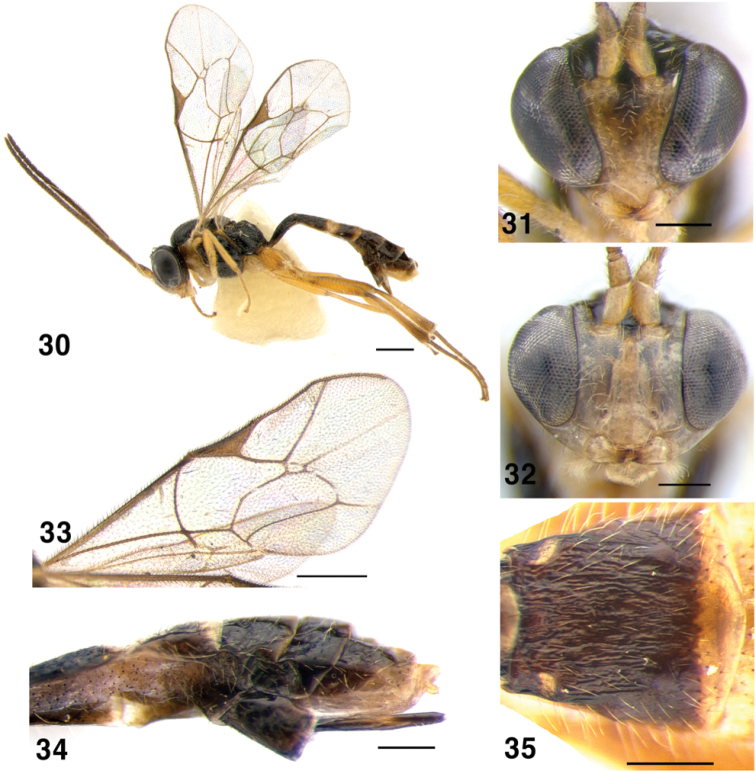
*Symplecis bicingulata*; **30** Habitus, lateral view **31** Head of female, anterior view **32** Head of male, anterior view **33** Wings **34** Metasoma, distal tergites, lateral view and ovipositor **35** Metasoma, second tergite, dorsal view. Scale bars: 0.5 mm (**30, 33**); 0.2 mm (**31, 32, 34, 35**).

**Figures 36–41.**
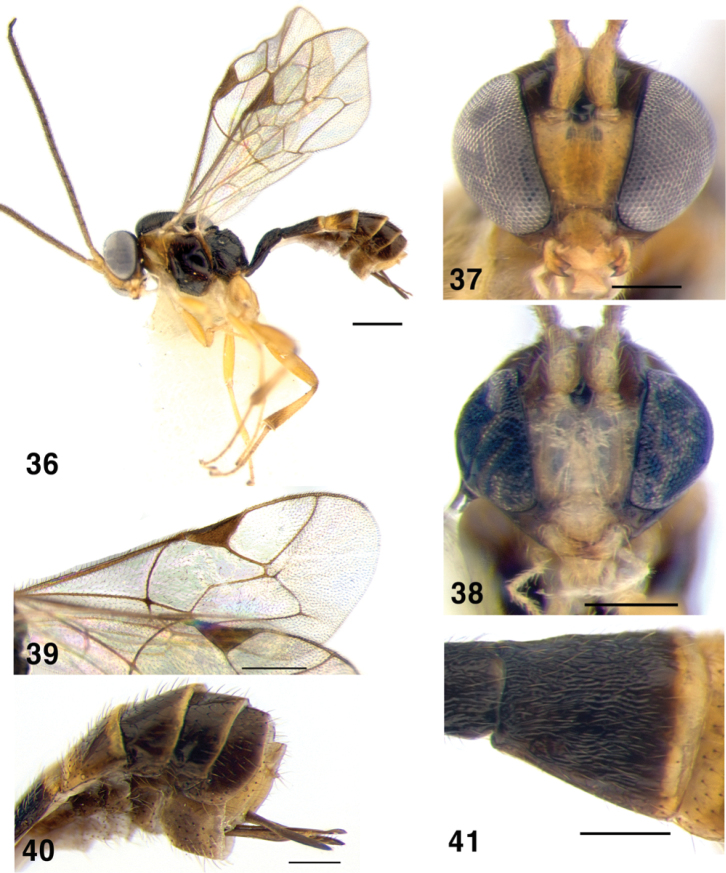
*Symplecis invisitata*; **36** Habitus, lateral view **37** Head of female, anterior view **38** Head of male, anterior view **39** Wings **40** Metasoma, distal tergites, lateral view and ovipositor **41** Metasoma, second tergite, dorsal view. Scale bars: 0.5 mm (**36, 39**); 0.2 mm (**37, 38, 40, 41**).

#### Symplecis bicingulata

Taxon classificationAnimaliaHymenopteraIchneumonidae

(Gravenhorst, 1829)

Figs 30–35

Mesoleptus bicingulatus Gravenhorst, 1829: 107; type depository: IZU.

##### Diagnosis.

Inner eye orbits strongly convergent ventrally in female (Fig. 31), face at the level of clypeus 0.41–0.45 times as wide as at the level of median ocellus; slightly convergent in male (Fig. 32). Mesoscutum polished; sternaulus short and deep; epomia lacking. Area superomedia of propodeum slightly transverse. Fore wing with areolet (Fig. 33). First metasomal tergite matt and striate, dorsal carina well developed; second tergite with distinct longitudinal striae (Fig. 35). Ovipositor short, hardly surpassing apex of metasoma (Fig. 34). Face and pronotum usually yellowish; tergites 2–3 apically and 3–4 basally yellow, forming two light bands on dark metasoma (Fig. 30).

##### Material examined.

Korea: 10 females, GW, Donghae-si, Samhwa-dong, Mureung Valley, [9–17 August 2005, 22 June–3 July 2006, 17–28 August 2006, 10–20 September 2006, 20 September–2 October 2006, 9 November 2006] (J.W. Lee); 1 female, ditto, 28 August–9 October 2006 (K.B. Kim); 1 male, GW, Wonju-si, Heungeup-myeon, Yeonse Univ., 22 July–11 August 2007 (J.W. Lee); 2 females, GG, Anyang-si, Manan-gu, Anyang-dong, Kwanagsan, 15–25 July 2008 (J.O. Lim); 1 male, GB, Gyeongsan-si, Daehak-ro, Yeungnam Univ., 22 April–1 May 2006 (J.W. Lee); 1 male, Ulsan-si, Sangbuk-myeon, Gajisan, 11 August 1989 (J.W. Lee).

##### Distribution.

Holarctic; in Palaearctic region reported from Europe, Siberia, Russian Far East (Primorsky Terr., Sakhalin Isl., Kunashir Isl.) (Humala 2007) and Japan (Dasch 1992; no data on the examined material was provided) and South Korea (new record).

#### Symplecis invisitata

Taxon classificationAnimaliaHymenopteraIchneumonidae

Rossem, 1981

Figs 36–41

Symplecis invisitata Rossem, 1981: 126–127; type depository: AEI.

##### Diagnosis.

Inner eyes orbits strongly convergent ventrally in both sexes (Figs 37, 38), malar space very narrow. Mesoscutum coriaceous, densely punctate; epomia present. Sternaulus shallow. Area superomedia of propodeum longer than wide. Fore wing without areolet (vein 3rs-m absent) (Fig. 39). First metasomal tergite coriaceous, dorsal carina obsolete; second tergite coriaceous (Fig. 41). Ovipositor long, 0.8–0.95 times as long as hind tibia. Second tergite fuscous with yellow posterior band, third tergite mostly yellow with transverse fuscous band in posterior half (Fig. 36).

##### Material examined.

Korea: 1 female, GW, Donghae-si, Samhwa-dong, Mureung Valley, 9–17 August 2005, (J.W. Lee); 1 male, GW, Hongcheon-gun, Hongcheon-eup, Jangjeoonpyeong-ri, Geodungae village, 1–14 July 2006, (J.W. Lee); 1 female, CB, Chungju-si, Suanbo-myeon, Samun-ri, Mt. Woraksan, 35°49'46"N, 128°04'05"E, Malaise trap, 17 July–12 August 2013, (J.K. Choi) (YNU).

##### Distribution.

Holarctic; reported in the Eastern Palaearctic from Kamchatka Peninsula, Primorsky Terr., Sakhalin Isl. (Humala 2007) and South Korea (new record).

## Discussion

The fauna of Orthocentrinae of the Eastern Palaearctic and Oriental regions has been extremely poorly studied. There are only six known species of orthocentrine in China and four species in Japan (Yu et al. 2012); nine more species, omitted by Yu et al. (2012), were recorded in Japan resulting from the treatment of Russian collections stored at the Zoological Institute RAS (Humala 2007). In the Catalogue of Ichneumonidae of Russian Far East (Kasparyan et al. 2012) there are 28 genera and 110 species of Orthocentrinae *sensu* Humala (including Microleptinae, Cylloceriinae and Diacritinae), though the fauna has been insufficiently studied in the region.

Korean orthocentrines are very poorly known. We have been conducting an inventory of this subfamily since 2014. Up to now only two species of Orthocentrinae from the genera *Proclitus* Förster, 1869 and *Eusterinx* Förster, 1869 were reported from South Korea (Choi et al. 2014). Besides these genera and two genera reviewed in this publication, during preliminary sorting of large orthocentrine collections stored at the Yeungnam University, thirteen more genera have been found to occur in South Korea, namely *Orthocentrus*, *Picrostigeus* Förster, *Stenomacrus* Förster, *Batakomacrus* Kolarov, *Plectiscus* Gravenhorst, *Neurateles* Ratzeburg, *Apoclima* Förster, *Pantisarthrus* Förster, *Aperileptus* Förster, *Dialipsis* Förster, *Plectiscidea* Viereck, *Helictes* Haliday, and *Megastylus* Schiødte. The consequent treatment of these materials is planned. All listed 17 genera found by us in South Korea are entirely or predominantly Holarctic and many of them are abundant and species-rich within temperate zones of the Palaearctic. Taking into account that the orthocentrine fauna of Japan and China is practically unstudied, the generic composition of the South Korean Orthocentrinae fauna could be compared with that of the Russian Far East, which contains approximately twice as many genera as Korea (eight Palaearctic genera: *Aniseres* Förster, *Atabulus* Rossem, *Entypoma* Förster, *Fennomacrus* Humala, *Hemiphanes* Förster, *Proeliator* Rossem, *Catastenus* Förster and *Terminator* Humala are not registered there). Most Korean species also occur in the Russian Far East (Humala 2007, Kasparyan et al. 2012). However some new species in the genera *Megastylus*, *Eusterinx*, *Plectiscus* and *Orthocentrus* have been discovered already, and they will be described in our forthcoming papers, devoted to the Korean fauna of the subfamily.

## Supplementary Material

XML Treatment for Gnathochorisis

XML Treatment for Gnathochorisis fuscipes

XML Treatment for Gnathochorisis koreensis

XML Treatment for Gnathochorisis crassulus

XML Treatment for Gnathochorisis dentifer

XML Treatment for Gnathochorisis flavipes

XML Treatment for Symplecis

XML Treatment for Symplecis bicingulata

XML Treatment for Symplecis invisitata
